# Steroid Sparing Maintenance Immunosuppression in Highly Sensitised Patients Receiving Alemtuzumab Induction

**DOI:** 10.3389/ti.2023.11056

**Published:** 2023-06-02

**Authors:** Eva Santos, Katrina Spensley, Nicola Gunby, Candice Clarke, Arthi Anand, Candice Roufosse, Michelle Willicombe

**Affiliations:** ^1^ Histocompatibility and Immunogenetics Laboratory, Northwest London Pathology NHS Trust, Hammersmith Hospital, London, United Kingdom; ^2^ Imperial College Renal and Transplant Centre, Imperial College Healthcare NHS Trust, Hammersmith Hospital, London, United Kingdom; ^3^ Centre for Inflammatory Disease, Department of Immunology and Inflammation, Imperial College London, Hammersmith Campus, London, United Kingdom; ^4^ Department of Histopathology, Northwest London Pathology NHS Trust, Charing Cross Hospital, London, United Kingdom

**Keywords:** HLA, highly sensitised, Alemtuzumab, calculated reaction frequency, steroid sparing

## Abstract

This analysis reports on the outcomes of two different steroid sparing immunosuppression protocols used in the management of 120 highly sensitised patients (HSPs) with cRF>85% receiving Alemtuzumab induction, 53 maintained on tacrolimus (FK) monotherapy and 67 tacrolimus plus mycophenolate mofetil (FK + MMF). There was no difference in the median cRF or mode of sensitisation between the two groups, although the FK + MMF cohort received more poorly matched grafts. There was no difference in one-year patient or allograft survival, however rejection free survival was inferior with FK monotherapy compared with FK + MMF at 65.4% and 91.4% respectively, *p* < 0.01. DSA-free survival was comparable. Whilst there was no difference in rates of BK between the cohorts, CMV-free survival was inferior in the FK + MMF group at 86.0% compared with 98.1% in the FK group, *p* = 0.026. One-year post-transplant diabetes free survival was 89.6% and 100.0% in the FK and FK + MMF group respectively, *p* = 0.027, the difference attributed to the use of prednisolone to treat rejection in the FK cohort, *p* = 0.006. We report good outcomes in HSPs utilising a steroid sparing protocol with Alemtuzumab induction and FK + MMF maintenance and provide granular data on immunological and infectious complications to inform steroid avoidance in these patient groups.

## Graphical Abstract

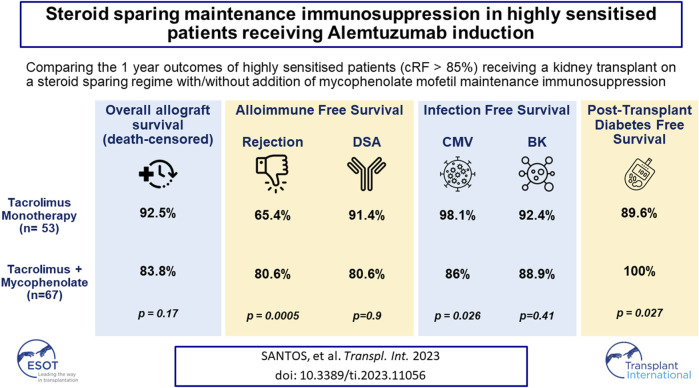

## Introduction

Lymphocyte-depleting induction therapy enables the use of steroid sparing immunosuppression protocols in kidney transplantation. In immunologically high-risk transplant recipients, Alemtuzumab induction has been shown to be equivalent to anti-thymocyte globulin (ATG) in preventing acute rejection in the first-year post-transplant in patients following early steroid withdrawal [1].

In the absence of a positive crossmatch, the definition of immunological risk is unclear, and there remains no uniform consensus on the clinical relevance of preformed DSA detected by single antigen beads (SAB) in the context of a negative crossmatch [2, 3]. Paradoxically, SABs are used to define sensitisation status *via* the calculation reaction frequency (cRF), which is often used to identify “patients at immunological risk” and guide immunosuppression regimens [1, 4–6]. Unquestionably, highly sensitised patients wait longer for an appropriate donor, but in the absence of a detectable DSA by SAB, the contribution of preformed HLA antibodies to post-transplant associated alloimmune injury is not as clear [4, 5, 7].

The 2019 Kidney Offering Scheme (KOS2019) in the UK was implemented to try and improve equity in access to transplantation. Under the scheme, highly sensitised patients (defined as those patients with a cRF≥85%), patients with difficult to match HLA types and long waiters (>7 years) were given allocation priority since its inception in September 2019. In preparation for the increased rates of highly sensitised patients receiving transplants, there was a change to our centre’s immunosuppression protocol, which included the addition of mycophenolate mofetil (MMF) to maintenance tacrolimus (FK) following Alemtuzumab induction (FK + MMF), together with early steroid withdrawal in all patients with a cRF≥85%.

The aim of this report is to investigate the clinical outcomes, both immunological and infectious, in transplant recipients with a cRF≥85% receiving Alemtuzumab induction with FK + MMF compared with FK monotherapy.

## Methods and Materials

### Patients

Patients transplanted at a single centre were identified from a prospectively maintained transplant registry. All patients transplanted from 2014 to 2022 were selected if they met the following criteria: cRF≥85% at the time of transplantation, had no identifiable preformed DSA (by SAB or positive crossmatch), received an ABO compatible transplant, had primary function and received Alemtuzumab induction. This study was approved by the West of Scotland Research Ethics Committee (20/WS/0181), and was performed in accordance with the Declaration of Helsinki and UK Data Protection legislation.

### UKT Matching Definitions

This paper will refer to HLA mismatch levels and matchability score. The mismatch level defines the antigen mismatches at HLA-A, HLA-B and HLA-DR. It is on a scale of 1-4, as defined by NHS Blood and Transplant [8]: Level 1 representing a 000 antigen match; Level 2 any single HLA mismatch at HLA-B or HLA-DR ([0 DR and 0/1 B] or [1 DR and 0 B]); Level 3 mismatch, a two antigen HLA-B mismatch or single mismatch at HLA-B plus HLA-DR ([0 DR and 2 B] or [1 DR and 1 B]) and a Level 4 mismatch representing either a single HLA-DR plus 2 HLA-B mismatches or two HLA-DR mismatches ([1 DR and 2 B] or [2 DR]). The matchability score is defined by ODT as a measure of how difficult it is to match a patient with an organ donor in the UK, based on comparison with a pool of 10,000 donor HLA types on a national database [8]. Matchability is defined on a scale of 1–10, 1–3 representing easy matchability, 4–7 medium matchability and 8–10 a difficult matchability, in patients with rare HLA types.

### Immunosuppression Protocol

The immunosuppression protocol consists of 0.4 mg/kg of alemtuzumab (Campath 1H, Genzyme, Oxford, UK) in the immediate post-operative period. All patients received methylprednisolone 500 mg pre-operative, followed only by a one-week course of corticosteroids. All patients received tacrolimus, with the observed cohort receiving maintenance mycophenolate mofetil (MMF) in addition. The target trough levels are 6–8 ng/mL for tacrolimus and 1.2–2.4 mg/L for mycophenolate.

### HLA Typing and DSA Monitoring

From June 2020, all donors and recipients were typed using high resolution next generation sequencing using GenDx MX6-1 HLA typing kits (HLA-A, -B, -C, -DRB1, -DQB1 and -DPB1). Sequencing reaction was performed using the Illumina iSeq™ platform and results analysed on GenDx NGS engine. All other recipients and transplant donors were routinely typed for HLA‐A, HLA‐B, HLA‐C, HLA‐DRB1/B3/B4/B5, and HLA‐DQB1 loci using in house PCR‐SSP (sequence‐specific primers) or the LABType® SSO typing kits (One Lambda ThermoFisher Scientific Inc., Canoga Park, CA, USA). Additional typing, e.g., DPA, DPB and DQA is performed retrospectively in the setting of *de novo* HLA antibodies.

Crossmatching was performed by T and B cell complement dependent cytotoxicity (CDC) and T-cell flow cytometry (FCXM) techniques, together with a single antigen screen. Transplants from donors where the recipient is known to have a preformed DSA detected by SAB are not routinely permissible [9].

Post-transplant, DSA are detected either as part of a screening protocol or at times of allograft dysfunction. Protocolised screening occurs twice in the first week, at 1 month, 3 months and 12 months. Screening is performed using LABScreen mixed beads (One Lambda, Canoga Park, CA) if the patient is non-sensitised and then subsequently or primarily screened using LABScreen single antigen beads if sensitised. Samples were treated with ethylenediaminetetraacetic acid (EDTA) to avoid possible prozone effect and the antibody pattern was interpreted taking into account the patient’s own HLA type. A mean fluorescence intensity (MFI) value of >1,000 by single antigen beads on two separate occasions was considered positive for the presence of antibody.

### Indications for Biopsy

Patients with a newly detected DSA, are offered a protocol biopsy unless there is a contraindication. Patients are also offered biopsies at times of allograft dysfunction. All rejection episodes were biopsy proven, unless otherwise stated. Biopsy-proven rejection episodes were defined using the 2019 Banff classification for Allograft Pathology, including cases borderline for T-cell mediated rejection (TCMR) and cases of chronic active TCMR. The 2013 Banff definitions were used to also include cases that showed histological features suspicious for active and chronic active antibody-mediated rejection, and cases that were C4d-positive without other features of rejection. Patients receiving tacrolimus monotherapy who had a biopsy for a DSA in the setting of stable allograft function had augmentation in their immunosuppression, with the addition of MMF, even in the absence of rejection. Patients who had subclinical rejection in the context of a DSA were treated with corticosteroids in addition to tacrolimus and MMF. Patients with antibody mediated rejection (ABMR) in the context of a newly detected DSA and allograft dysfunction were treated with plasma exchange and intravenous immunoglobulin, in addition to the introduction of corticosteroids. Patients who have a DSA detected in the first 14 days post-transplant were treated for presumed ABMR in the context of graft dysfunction.

### Detection and Diagnosis of Immunosuppression Complications

For the purposes of this analysis, the following definitions were used to define complications associated with immunosuppression. Post-transplant diabetes was defined as the *de novo* need for hypoglycaemic agents (oral or insulin) in the follow up period. A diagnosis of BK viraemia was made on the detection of BK virus DNA in 2 or more blood samples by PCR testing. A diagnosis of CMV viraemia was similarly made *via* PCR testing of 2 separate blood samples.

### Statistical Analysis

All analyses were performed using GraphPad Prism version 9.4.0. Comparisons of means and frequencies of normally distributed variables were calculated using t-tests and chi-square/Fisher’s exact tests. The Mann–Whitney test was used for nonparametric variables. The Kaplan–Meier estimator was used for survival analysis related to clinical outcome following transplantation; statistical significance was determined by log rank testing. Multivariate analyses were calculated using Cox proportional hazards regression models. A *p*-value of <0.05 was deemed statistically significant.

## Results

**FIGURE 1:**
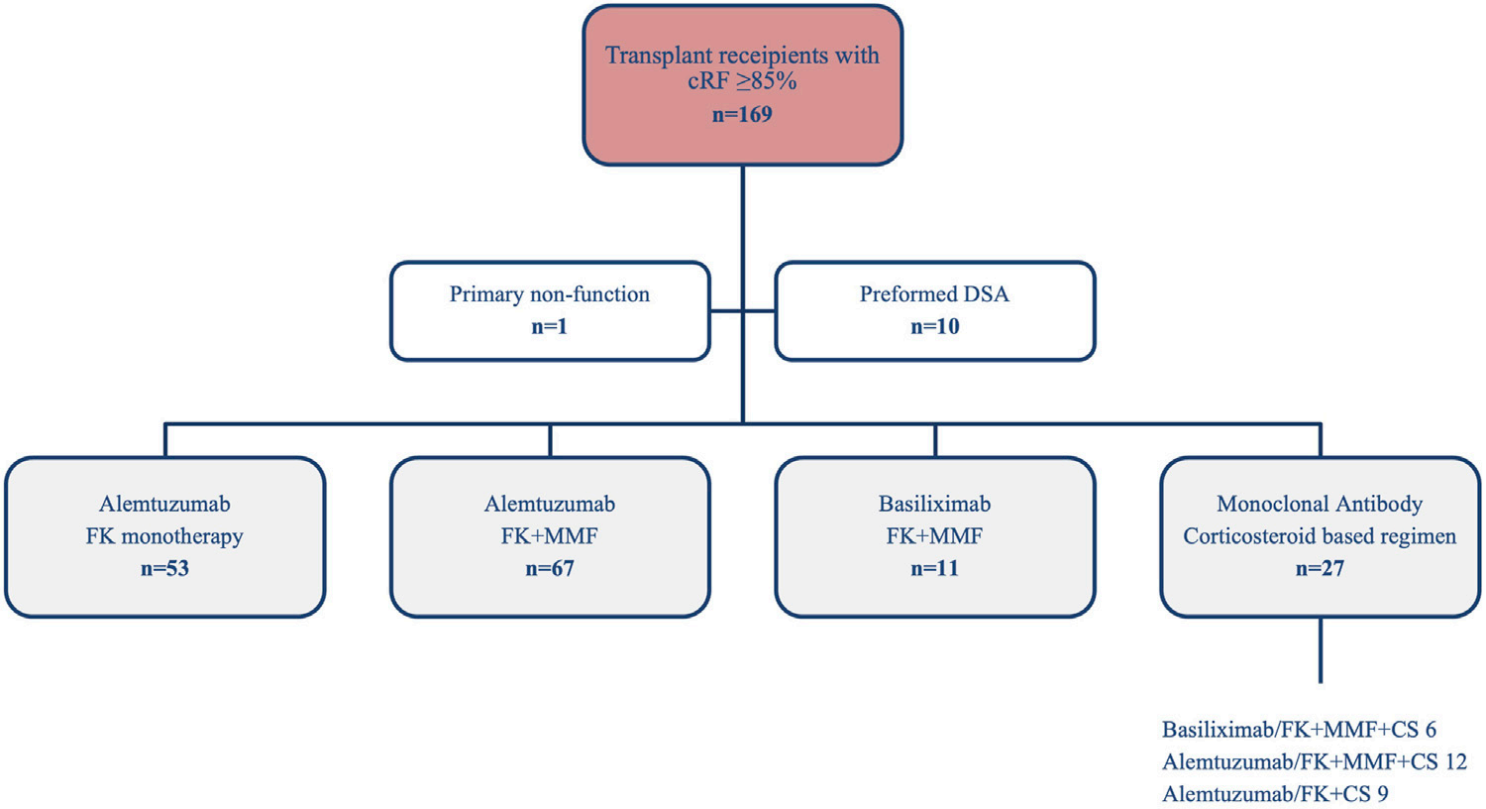
Highly sensitised (cRF≥85%) recipients transplanted between 2014 and 2022 by immunosuppression regimen.

One-hundred and sixty-nine highly sensitised patients (HSPs) were identified over the analysis period. Fifty-three (31.4%) patients received Alemtuzumab plus FK monotherapy and 67 (39.6%) received Alemtuzmab plus FK + MMF. In addition, 11 (6.5%) received Basiliximab plus FK + MMF, 27 (16.0%) received either Alemtuzumab or Basiliximab with prednisolone based maintenance therapy, and an additional 11 (6.5%) patients were excluded (1 primary non-function and 10 preformed DSA), Figure 1. For comparison of the clinical outcomes for the different immunosuppression regimes see Supplementary Materials. From here, we will report on patients receiving Alemtuzumab induction and tacrolimus either with or without MMF, Table 1.

**TABLE 1:** Characteristics of highly sensitised patients receiving Alemtuzumab.

		FK monotherapy	FK + MMF	*p*-value
		*n* = 53 (%)	*n* = 67 (%)	
Gender	Female	40 (75.5)	46 (68.7)	0.41
	Male	13 (24.5)	21 (31.3)	
Age at Transplant	Years (median)	54 (46–59)	54 (45–62)	0.62
Ethnicity	Black	11 (20.8)	13 (19.4)	0.98
	Caucasian	11 (20.8)	16 (23.9)	
	Indoasian	23 (43.4)	28 (41.8)	
	Other	8 (15.1)	10 (14.9)	
Cause of ESRD	APKD	4 (7.5)	5 (7.5)	0.39
	DM	6 (11.3)	14 (20.9)	
	GN	10 (18.9)	19 (28.4)	
	Other	7 (13.2)	5 (7.5)	
	Unknown	22 (41.5)	19 (28.4)	
	Urological	4 (7.5)	5 (7.5)	
Diabetes	No	40 (75.5)	48 (71.6)	0.64
	Yes	13 (24.5)	19 (28.4)	
Pre-emptive transplant	No	51 (96.2)	64 (95.5)	0.85
	Yes	2 (3.8)	3 (4.5)	
Time on wait list	Days (Median)	1,711 (974–2,599)	1,523 (1,092–2,343)	0.86
Donor Type	LD	16 (30.2)	4 (6.0)	0.0004^a^
	DD	37 (69.8)	63 (94.0)	
	DBD	30 (81.1)	40 (63.5)	0.065
	DCD	7 (18.9)	23 (36.5)	
Cold Ischaemic Time (deceased donors)	Hours (median)	11.3 (9.3–14.5)	12.0 (9.2–14.0)	0.57
Delayed Graft Function	No	48 (90.6)	40 (59.7)	0.0002^a^
	Yes	5 (9.4)	27 (40.3)	

^a^ *p* < 0.05.

### Comparison of Baseline Patient Characteristics

There was no gender difference between those recipients who received FK compared with FK + MMF, with 40/53 (75.5%) and 46/67 (68.7%) respectively being female, *p* = 0.41, Table 1. There was no difference in median age at the time of transplant, at 54 (46–59) and 54 (45–62) years in the FK and FK + MMF groups respectively, *p* = 0.62. There was also no proportional difference in ethnicity distribution, with 11/53 (20.8%) and 16/67 (23.9%) patients in the FK and FK + MMF groups being from a white ethnic background respectively, *p* = 0.69. Pre-emptive transplant rates were low overall, with no difference in the FK and FK + MMF groups at 2/53 (3.8%) and 3/67 (4.5%) respectively, *p* = 0.085. The corresponding time on the wait list was long, with a median time to transplant of 1711 (974–2,599) days in patients receiving FK and 1,523 (1,092–2,343) days in patients who received FK + MMF, *p* = 0.86. The proportion of patients who received a living donor kidney was significantly higher in the FK group at 16/53 (30.2%) compared with 4/67 (6.0%) in the patients who received FK + MMF, *p* = 0.0004. There was neither a difference in the median cold ischaemic time, 11.3 (9.3–14.5) hours and 12.0 (9.2–14.0) hours in the FK and FK + MMF groups, *p* = 0.57; or a difference in the deceased donor type, with 7 (18.9%) and 23 (36.5%) patients receiving transplants from donors after cardiac death in the FK and FK + MMF groups respectively, *p* = 0.065. However, the proportion of patients who experience delayed graft function, was significantly lower in the group receiving FK, 5/53 (9.4%), compared with FK + MMF, 27 (40.3%), *p* = 0.0002.

### Comparison of Baseline Immunological Characteristics

The median cRF in the FK and FK + MMF groups was 96 (90–99)% and 98 (94–99)%, *p* = 0.45, with 8/53 (15.1%) and 15/67 (22.4%) patients having a cRF of 100% respectively, *p* = 0.32, Table 2. Pregnancy was the leading mode of sensitisation in both groups, with 37/53 (69.8%) and 39/67 (58.2%) patients in the FK and FK + MMF cohorts respectively having pregnancy contribute to their highly sensitised status, *p* = 0.19. Whilst in the FK and FK + MMF cohorts, 12/53 (22.6%) and 21/67 (31.3%) respectively were receiving at least a second solid organ transplant, *p* = 0.29; with 2/12 (16.7%) and 3/21 (14.3%) receiving an organ with a repeat HLA mismatch, *p* = 0.86.

**TABLE 2:** Immunological Characteristics of highly sensitised patients receiving Alemtuzumab.

		FK monotherapy	FK + MMF	*p*-value
		*n* = 53 (%)	*n* = 67 (%)	
cRF Group	85%–94%	19 (35.8)	18 (26.9)	0.45
	95%–99%	26 (49.1)	34 (50.7)	
	100%	8 (15.1)	15 (22.4)	
cRF	% (Median)	96 (90–99)	98 (94–99)	0.087
Route of sensitisation	Blood	6 (11.3)	12 (17.9)	0.38
	Pregnancy ± Blood	35 (66.0)	34 (50.7)	
	Tx ±Blood	10 (18.9)	16 (23.9)	
	Pregnancy, Tx ±Blood	2 (3.8)	5 (7.5)	
Blood product transfusion in 1st 28 days post-transplant	Yes	22 (41.5)	25 (37.3)	0.64
	No	31 (58.5)	42 (62.7)	
Live Donor-Recipient Relationship	Unrelated	8 (15.1)	2 (3.0)	0.23
	Sibling	4 (7.5)	0	
	Child to Mother	2 (3.8)	0	
	Child to Father	1 (1.9)	0	
	Parent to Child	1 (1.9)	1 (1.5)	
	Partner (Male to Female)	0	1 (1.5)	
Repeat transplant	No	41 (77.4)	46 (68.7)	0.29
	Yes	12 (22.6)	21 (31.3)	
Repeat HLA mismatch	Yes	2 (3.8)	3 (6.4)	0.55
Matchability	Easy	-	2 (3.0)	0.15
	Medium	13 (24.5)	9 (13.4)	
	Difficulty	40 (75.5)	56 (83.6)	
Matchability score	Median	9 (7.75–10)	9 (8–10)	0.16
Total ABDR MM	Median	3 (2–4)	3 (3–4)	0.10
UKT MM Level^a^	1	4 (7.5)	1 (1.5)	0.03
	2	17 (32.1)	15 (22.4)	
	3	13 (24.5)	17 (25.4)	
	4	19 (35.8)	34 (50.7)	
HLA A Mismatch	0	11 (20.8)	12 (17.9)	0.59
	1	26 (49.1)	32 (47.8)	
	2	16 (30.2)	23 (34.3)	
HLA B Mismatch	0	14 (26.4)	7 (10.4)	0.04
	1	24 (45.3)	30 (44.8)	
	2	15 (28.3)	30 (44.8)	
HLA Cw Mismatch	0	13 (24.5)	11 (16.4)	0.29
	1	30 (56.6)	36 (53.7)	
	2	10 (18.9)	20 (26.9)	
HLA DRB1 Mismatch	0	16 (30.2)	17 (25.4)	0.62
	1	28 (52.8)	34 (50.7)	
	2	9 (17.0)	16 (23.9)	
HLA DQB1 Mismatch	0	23 (43.4)	25 (37.3)	0.79
	1	24 (45.3)	34 (50.7)	
	2	6 (11.3)	8 (11.9)	

^a^ Using KOS 2019 mismatch level.

Overall, the majority of patients, 96/120 (80.0%), had a HLA type that was recognised as “difficult” to match, with no proportional difference in the FK and FK + MMF groups, *p* = 0.15, Table 2. There was no difference in the ABDR mismatch between those who received FK, with a median 3 (IQR 2–4) mismatches, compared with those who received FK + MMF, with a median 3 (IQR 3–4) mismatches, *p* = 0.10. However, patients who received FK monotherapy were less likely to receive an unfavourable Level UKT mismatch compared with those receiving FK + MMF, with 19/53 (35.8%) and 34/67 (50.7%) receiving a Level 4 [2DR or (1DR+1B)] mismatched kidney respectively, *p* = 0.03, Table 2. Whilst there was no difference in overall mismatches at HLA-A (*p* = 0.59), HLA-Cw (*p* = 0.29), HLA-DRB1 (*p* = 0.62) and HLA-DQB1 (*p* = 0.79) between the FK and FK + MMF cohorts, patients receiving FK were more likely to receive a kidney matched at HLA-B compared with those patients who received FK + MMF, *p* = 0.023.

### Comparison of Clinical Outcomes

The overall median follow up for all patients was 2.8 (1.6–5.1) years, with longer follow up in FK monotherapy group at 5.5 (4.4–6.8) years compared with 1.7 (0.9–2.5) years in the FK + MMF cohort, *p* < 0.0001. Clinical outcomes were therefore restricted to 1 year post-transplant.

**FIGURE 2:**
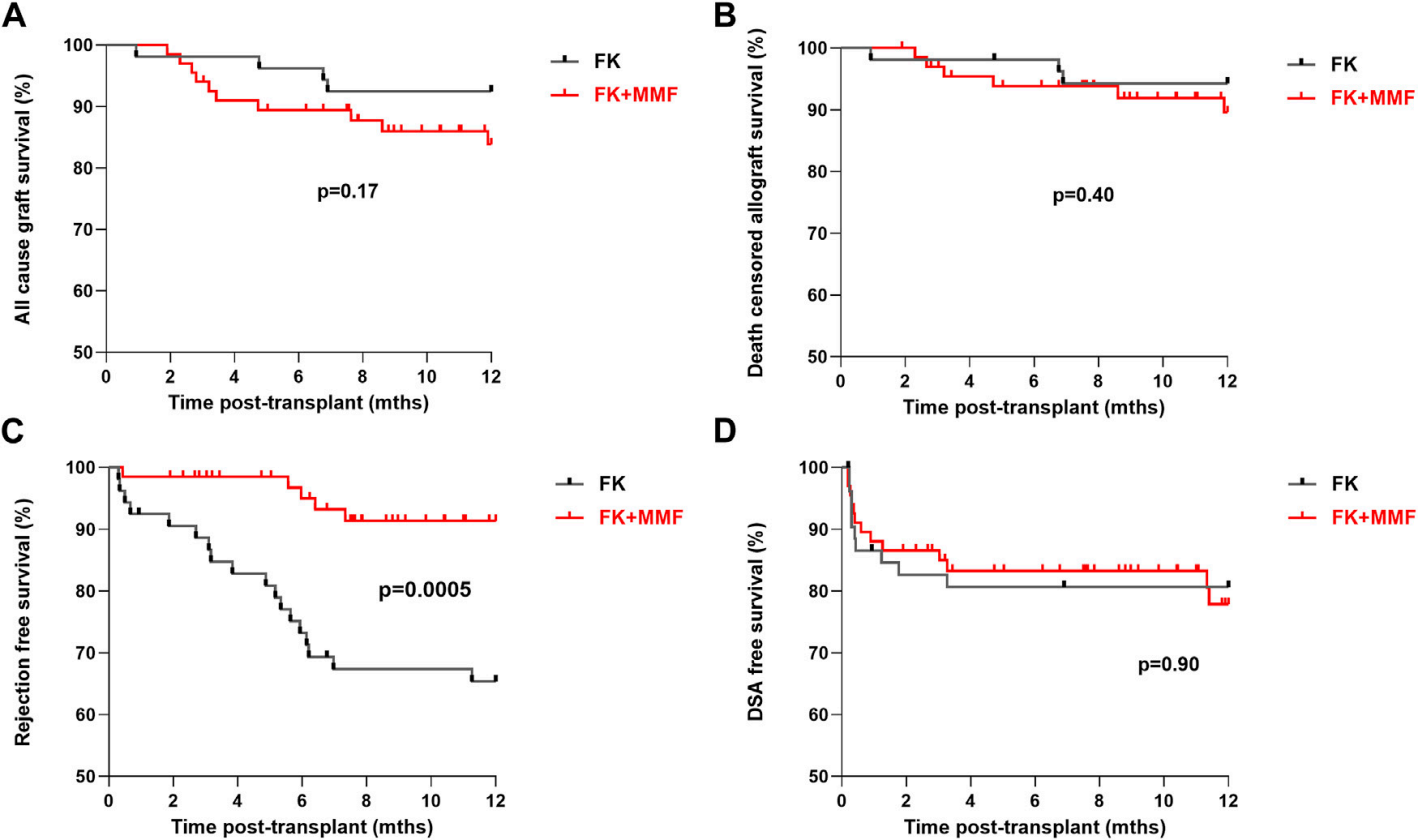
Clinical outcomes associated with and without the use of mycophenolate in highly sensitised patients **(A)** All-cause 1 year allograft survival was 92.5% and 83.8% in the FK and FK + MMF groups respectively, *p* = 0.17 (log-rank) **(B)** Death censored allograft survival was 94.3% and 89.6% in the FK and FK + MMF groups respectively, *p* = 0.40 (log-rank) **(C)** One-year rejection free survival was inferior in the FK cohort compared with the FK + MMF cohort at 65.4% and 91.4% respectively, *p* = 0.0005 (log-rank) **(D)** One-year DSA free survival was 80.6% and 80.6%, in the FK and FK + MMF cohort respectively, *p* = 0.90 (log-rank).

All-cause 1 year allograft survival was 92.5% and 83.8% in the FK and FK + MMF groups respectively, *p* = 0.17, Figure 2. Five patients died with a functioning graft in the first year post-transplant, one patient in the FK group died of COVID-19 infection, whilst 4 patients in the FK + MMF group (1 COVID-19 infection, 1 sepsis and 2 cardiac). Death censored allograft survival was 94.3% and 89.6% in the FK and FK + MMF groups respectively, *p* = 0.40. There were 3 graft losses in the FK group (1 rejection, 1 transplant renal artery stenosis and 1 BK nephropathy), whilst there were 6 graft losses in the FK + MMF group (2 rejection, 1 transplant renal artery stenosis and 3 donor derived pathology). At 3 and 12 months post-transplant, estimated GFR was not statistically different between the FK and FK + MMF groups, at 54 (IQR 44–67) mLs/min and 46 (34–62) mLs/min respectively, *p* = 0.09 at 3 months, and 57 (46–66) mLs/min and 51 (37–65) mLs/min, *p* = 0.16 at 12 months.

One-year rejection free survival was inferior in the FK cohort compared with the FK + MMF cohort at 65.4% and 91.4% respectively, *p* = 0.0005, Figure 2. There were 18 episodes of treated rejection in the FK cohort (12 active ABMR, 4 borderline TCMR, 1 Banff 1 TCMR and 1 case of C4d+ without evidence of rejection in the setting of graft dysfunction), and 5 cases in the FK + MMF cohort (3 active ABMR, 1 borderline TCMR and 1 presumed rejection in the context of a DSA plus acute allograft dysfunction).

One-year DSA free survival was comparable in the two groups, with a reported survival of 80.6% and 80.6%, or 10 and 12 patients with DSA in the FK and FK + MMF cohort respectively, *p* = 0.90. Fifteen of 22 (68.2%) patients, 7/10 (70.0%) and 8/12 (66.7%) patients in the FK and FK + MMF cohort respectively had the DSA detected in the first 30 days post-transplant, suggesting a likely memory response in the majority of cases. Six of the remaining seven DSA positive patients had the DSA detected between days 31 and the 3-month screen, 3 patients in each of the FK and FK + MMF groups. Only 1 patient developed a DSA between 3 and 12 months, this patient was in the FK + MMF cohort and had MMF stopped following a diagnosis of BK nephropathy. A high proportion of patients had a measured tacrolimus level below target, but there was no difference between the FK and FK + MMF groups; between 1 week and 3 months post-transplant, 33/53 (62.3%) and 36/67 (537%) patients respectively had at least one tacrolimus level below target, *p* = 0.35. Whilst between 3 months and 1 year, 30/52 (57.7%) of the FK group and 34/64 (53.1%) of the FK + MMF group had at least one tacrolimus level below target, *p* = 0.62.

**FIGURE 3:**
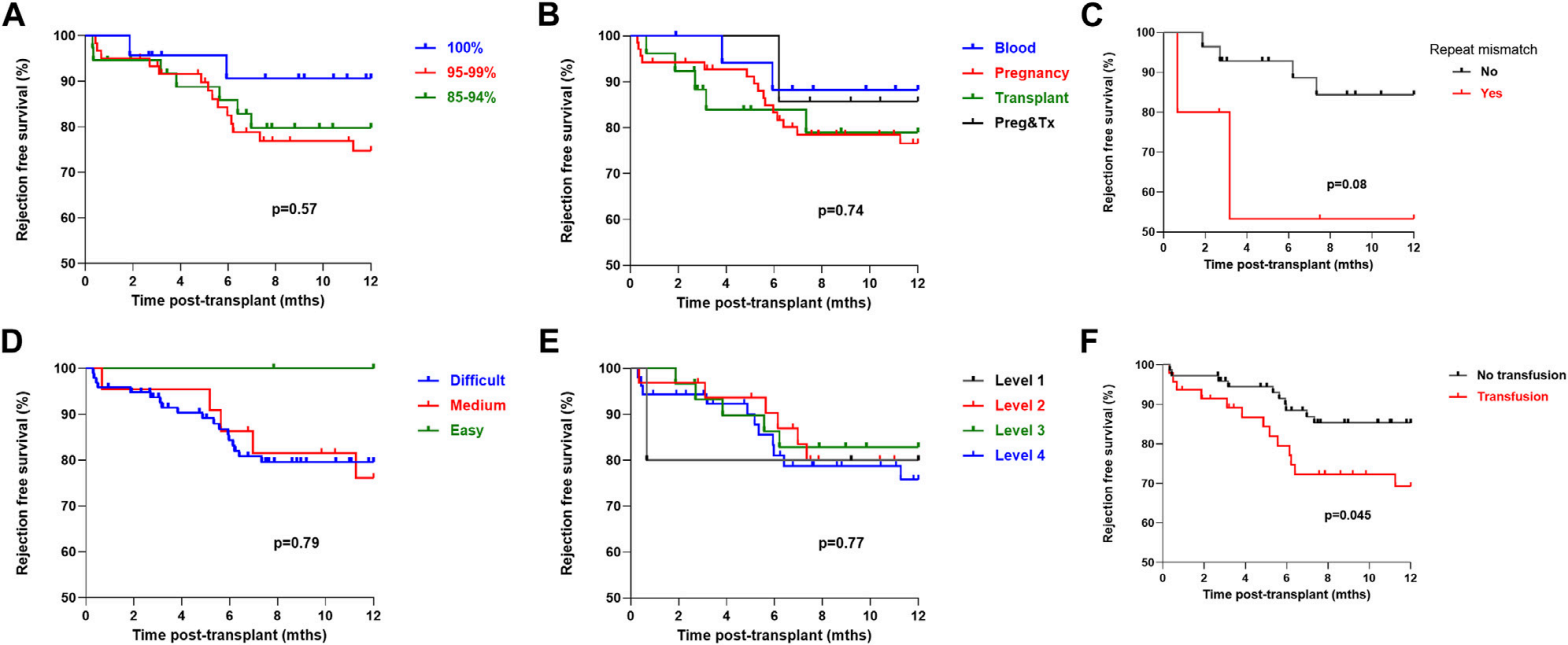
One-year rejection free survival by immunological characteristics (log-rank) One-year rejection free survival (log-rank) was **(A)** No difference by cRF status, *p* = 0.57 **(B)** No difference by mode of sensitisation, *p* = 0.74 **(C)** No difference in patients receiving a >2nd graft by presence or absence of repeat HLA mismatch, *p* = 0.08 **(D)** No difference by HLA matchability, *p* = 0.79 **(E)** No difference by UKT Level Mismatch, *p* = 0.77 **(F)** Inferior in patients who received a post-transplant blood transfusion, *p* = 0.045.

Investigating the role of immunological characteristics at the time of transplant with risk of rejection or DSA detection was performed next. On univariate analysis, cRF did not associate with likelihood of rejection in the first year post-transplant, with rejection free survival of 79.8%, 74.7% and 90.6% in the patients with a cRF of 85%–94%, 95%–99% and 100% respectively, *p* = 0.37, Figure 3. Mode of sensitisation also did not associate with rejection, with 1 year rejection free survival of 88.2%, 76.6%, 78.9% and 85.7% in patients with sensitised *via* blood, pregnancy, transplantation or pregnancy and transplantation respectively, *p* = 0.74. For the 33 patients receiving a ≥2nd transplant, a repeat HLA mismatch was not associated with risk of rejection, with a rejection free survival of 84.4% and 53.3% in those with and without re-exposure to a previous mismatched antigen, *p* = 0.08. Patients who received a blood product transfusion in the first 28 days post-transplant were at higher risk of rejection, with a one-year rejection free survival of 85.3% and 69.3% in those without and who had received a transfusion respectively, *p* = 0.045. There was no difference in risk of rejection according to UKT Level mismatch, with a one-year rejection free survival of 80.0%, 85.9%, 78.5% and 75.8% in patients receiving a Level 1,2,3 and 4 mismatch respectively, *p* = 0.77. One-year rejection free survival in patients with easy, medium and difficult to match HLA types was 100.0%, 76.1% and 79.6% respectively, *p* = 0.79.

**FIGURE 4:**
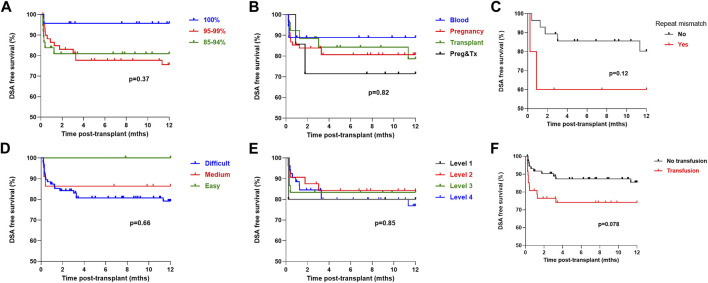
One-year DSA free survival by immunological characteristics One-year DSA free survival (log-rank) was **(A)** No difference by cRF status, *p* = 0.37 **(B)** No difference by mode of sensitisation, *p* = 0.82 **(C)** No difference in patients receiving a >2nd graft by presence or absence of repeat HLA mismatch, *p* = 0.12 **(D)** No difference by HLA matchability, *p* = 0.66 **(E)** No difference by UKT Level Mismatch, *p* = 0.85 **(F)** Inferior in patients who received a post-transplant blood transfusion, *p* = 0.078.

On univariate analysis, cRF did not associate with likelihood of a detectable DSA in the first-year post-transplant, with a DSA free survival of 81.0%, 75.5% and 95.7% in the patients with a cRF of 85%–94%, 95%–99% and 100% respectively, *p* = 0.37, Figure 4. Mode of sensitisation also did not associate with DSA, with 1 year DSA free survival of 88.9%, 80.7%, 84.2% and 71.4% in patients sensitised *via* blood, pregnancy, transplantation or pregnancy and transplantation respectively, *p* = 0.82. For the 33 patients receiving a ≥2nd transplant, a repeat HLA mismatch was not associated with risk of DSA, with a DSA free survival of 60.0% and 80.2% in those with and without re-exposure to a previous mismatched antigen, *p* = 0.12. Patients who received a blood product transfusion in the first 28 days post-transplant were not at statistically higher risk of a DSA, with a one-year DSA free survival of 85.5% and 74.1% in those without and who had received a transfusion respectively, *p* = 0.078. There was no difference in post-transplant DSA according to UKT Level mismatch, with a one-year DSA free survival of 80.0%, 85.9%, 87.3% and 77.2% in patients receiving a Level 1,2,3 and 4 mismatch respectively, *p* = 0.85. One-year DSA free survival in patients with easy, medium and difficult to match HLA types was 100.0%, 81.0% and 79.2% respectively, *p* = 0.66.

### Multivariate Analyses of Factors Associated With Allograft Loss, Rejection and DSA

Multivariate analysis of each outcome measure was performed, incorporating variables associated with corresponding outcome on univariate analysis. Factors associated with all-cause allograft loss included delayed graft function, HR 4.85 (1.57–14.98), *p* = 0.006 and total ABDR mismatch, HR 1.93 (1.18–3.14), *p* = 0.009. Whilst factors associated with censored allograft loss included delayed graft function, HR 5.90 (1.40–24.90), *p* = 0.016 and total ABDR mismatch, HR 1.83 (1.00–3.36), *p* = 0.049. Factors associated with rejection included receiving a graft across a repeat HLA mismatch, HR 9.50 (1.92–46.87), *p* = 0.006, and receiving FK monotherapy, HR 10.37 (2.80–38.33), *p* = 0.0005. Whilst no independent variables were found to be associated with risk of DSA at 1 year post-transplant.

### Comparison of Rates of the Adverse Effects of Immunosuppression

**FIGURE 5:**
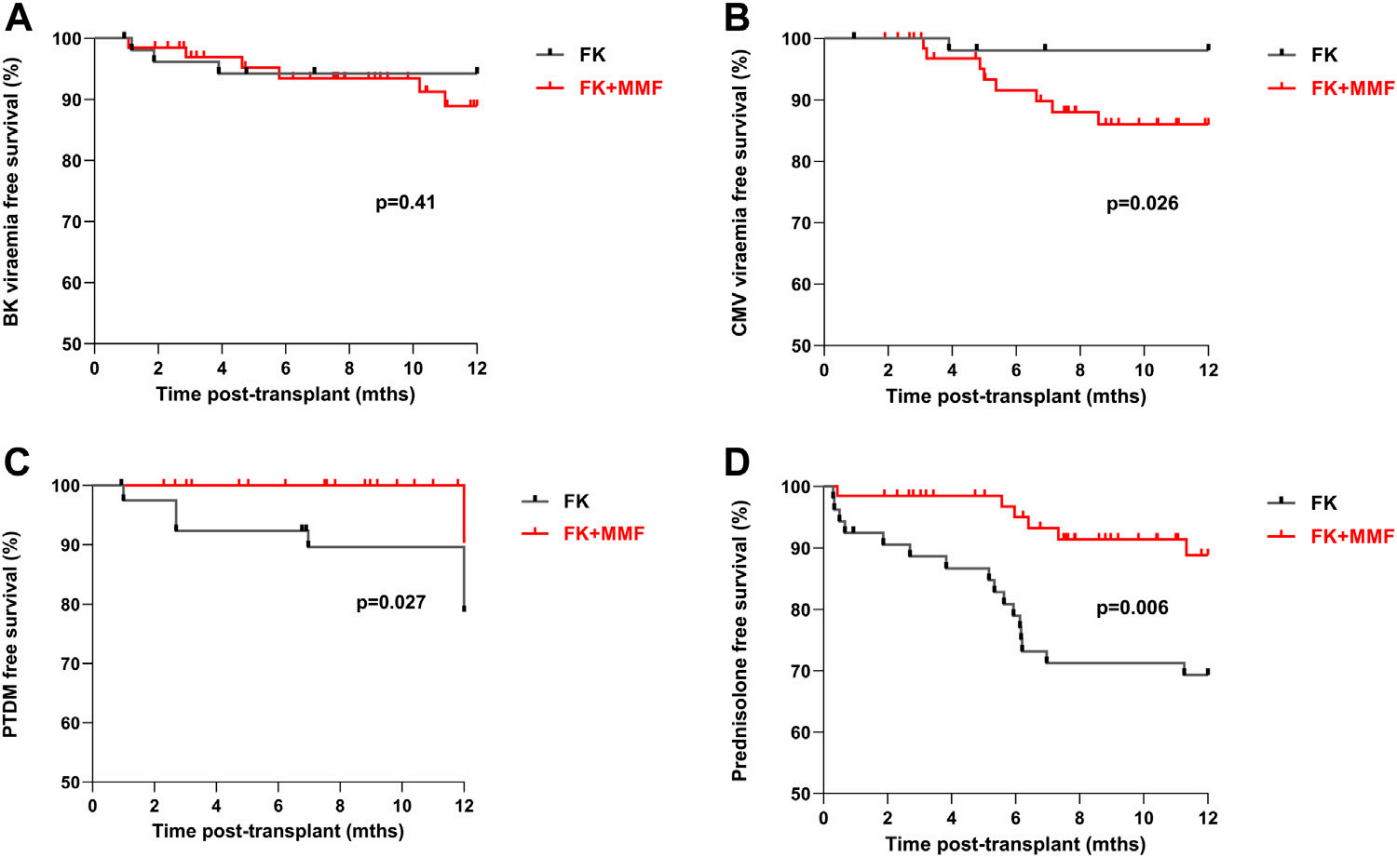
Adverse clinical outcomes associated with and without the use of mycophenolate in highly sensitised patients At one-year post-transplant, there was **(A)** No difference in BK-virus free survival between the FK and FK + MMF groups, *p* = 0.41 **(B)** Superior CMV free survival in the FK group compared with the FK + MMF group, *p* = 0.026 **(C)** Inferior PTDM free survival in the FK group compared with the FK + MMF group, *p* = 0.027 **(D)** Inferior prednisolone free survival in the FK group compared with the FK + MMF group, *p* = 0.006.

There was no significant difference in the one-year BK infection free survival in the FK monotherapy compared with the FK + MMF cohorts, at 94.2% and 88.9% respectively, *p* = 0.41, Figure 5. However, the one-year CMV free survival was superior in the FK monotherapy compared with the FK + MMF cohorts, at 98.1% and 86.0% respectively, *p* = 0.026. On multivariate analysis, total ABDR HLA mismatch was associated with increased risk of CMV, HR 1.99 (1.08–3.64), *p* = 0.03, whilst FK monotherapy was not significantly associated with a reduced risk statistically, they may be clinically relevant, HR 0.21 (0.04–1.05), *p* = 0.057. Crucially of note CMV risk by serological status of donor and recipient were not included in this model.

For patients not known to have diabetes at the time of transplant a new diagnosis of post-transplant diabetes (PTDM) was more likely in the FK monotherapy compared with the FK + MMF cohort, with a one-year PTDM free survival of 89.6% and 100.0% respectively, *p* = 0.027, Figure 5. The one-year prednisolone free survival in the FK monotherapy and FK + MMF cohorts being 69.3% and 88.8% respectively, *p* = 0.006.

## Discussion

In the absence of preformed DSA detected by SAB, highly sensitised patients at our centre have historically received our standard immunosuppression protocol of Alemtuzumab induction and tacrolimus monotherapy as maintenance. In this report, we have shown that this strategy results in comparable DSA detection, both memory and *de novo*, when compared with a similar immunosuppression protocol with the addition of MMF. However, overall rejection rates were much reduced in the latter cohort, suggesting under immunosuppression in the absence of MMF. Although we found increased CMV rates in patients receiving MMF, this adverse effect was counterbalanced by a higher incidence of post-transplant diabetes in the FK monotherapy cohort, which may be attributed to the introduction of corticosteroids following rejection in this group. This nicely demonstrates how any benefits of enhanced immunosuppression against rejection, may be offset by their metabolic and infectious complications.

Recent European guidelines on HLA sensitisation have summarised challenges surrounding the management of highly sensitised patients [10]. As highlighted in these guidelines, standardisation of definitions, alignment of allocation policies and harmonisation of treatment strategies are required to provide evidence for optimal management. An increasingly adopted definition of highly sensitised status is a cRF of ≥85%, determined by SAB methods. This cut-off has been utilised by Eurotransplant for many years, and more recently used in the KOS 2019 organ allocation scheme in the UK [8, 10, 11].

A high cRF associates with increasing difficulty in finding a compatible kidney, and hence such patients have to wait a longer time for a transplant. However, the additional immunological risk posed by a high cRF in the absence of preformed DSA and the impact on allograft survival is not clear. To date, there are conflicting reports. Huber et al. found cRF to be a significant risk factor for long-term graft survival in a study of 726 renal transplant recipients, although their conclusions were limited as they did not specify the absence of preformed DSA [6]. In contrast, Wehmeier et al. in a single centre study, found the broadness of sensitisation not to be an immunological risk factor for ABMR and graft loss [4]. In their study, the main pre-transplant risk factors were the presence or absence of preformed DSA, and the number of donor mismatches to which the recipient can develop post-transplant DSA [4]. This latter study has more recently been supported by registry data from the US, which concluded that cRF was a poor predictor of allograft outcomes, and only patients who were receiving repeat transplants with a cRF≥98% were at risk of death censored graft loss [5].

Despite the lack of consensus of cRF as a prognostic indicator for immunological risk, it is often used to guide induction immunosuppression [1, 10, 12]. This may be considered a pragmatic approach as pre-transplant sensitisation may help identify patients at risk of developing a memory response. Certainly, early DSA detection, suggesting a memory response was more common than *de novo* DSA in this report. In this case, peak or historic cRFs may more accurately correlate with memory, although dynamics may also work the other way, and for some patients, especially those awaiting regrafts, cRF may significantly increase on the wait-list over time [6, 13]. The inclusion of memory response testing as part of pre-transplant risk assessment would certainly be beneficial, although may be difficult to implement. The most common method available until now, HLA-ELISpot, is not suitable for routine use as it is time-consuming and not easily standardised. New Luminex based methods to test cultured B-cell supernatants, could be more promising for routine diagnostics. Although initially the sensitivity was low due to level of IgG, current modifications using concentrated, or IgG isolated supernatants are showing improved detection [14]. As part of wider immunological testing, assessment of pre-transplant T-cell immunity has also been proposed to help risk stratify patients, but is not yet incorporated into clinical use [15, 16].

Surprisingly, we did not find a significant association between matchability or UKT level mismatch on either rejection or DSA development in this highly sensitised cohort. This is in contrast with studies that have looked at the correlation between an increasing number of HLA antigen mismatches and alloimmune outcomes. In addition we have previously shown that higher HLA Level mismatches are associated with *de novo* DSA and ABMR [17, 18]. Our findings in this study, may relate to the possibility that degree of mismatch being less significant in transplants in highly sensitised patients, or it may be a reflection of the relatively small number of patients in our study.

This report supports the potential use of steroid sparing protocols in HSPs, with previous studies assessing efficacy of such protocols including sensitised patients down to a cRF≥20% [1, 19]. Whilst the patient outcomes in our report were impacted by COVID-19, assessing allograft outcomes alone, 1-year rejection free survival was excellent at 91.4% in the group receiving FK + MMF. This is reassuring as steroid sparing protocols are recognised to be associated with increased rates of acute rejection, but there are no reports of this translating into inferior graft survival [20]. Steroid sparing protocols may be of survival benefit in this population who may have accumulated significant co-morbidity whilst awaiting a transplant and are particularly attractive for our predominantly non-white population who are at increased risk of post-transplant diabetes [19, 21, 22].

This single centre report includes relatively small numbers with short-term follow up, which limit the power of its conclusions. However, larger studies on HSPs lack the granular data we report here on risk factors for memory response and DSA monitoring in HSPs lacking detectable DSA assessed by SAB and crossmatching. As transplant activity in HSPs remain at a high rate, we will continue to prospectively monitor and will report subsequently on patients we maintain on FK + MMF. Whilst the KOS2019 in the UK has led to an increase in the number of highly sensitised patients receiving transplants, as reflected in this report, this should not detract from the need to expand kidney sharing schemes and improve desensitisation protocols [22]. However, as a community we must do more to try and minimise sensitisation, with 2 modifiable areas requiring optimisation including preventing *de novo* sensitisation in patients returning to the wait-list post graft failure and prevention of sensitisation *via* blood product transfusion.

## Data Availability

The data analyzed in this study is restricted as it is clinical data. Requests to access these datasets should be directed to the corresponding author.
