# Supplementary material for: Steroid Sparing Maintenance Immunosuppression in Highly Sensitised Patients Receiving Alemtuzumab Induction
Source: Transpl Int. 2023 Jun 2;36:11056. doi: 10.3389/ti.2023.11056 (PMC10272412; doi:10.3389/ti.2023.11056)

**Supplemental Information**

**Comparison of different immunosuppression protocols in HSPs**

Compared with the FK monotherapy group, HSPs who received Basiliximab/FK+MMF, were not at lower risk of death-censored allograft loss (p=0.64), rejection (p=0.15) or DSA (p=0.125).

Compared with the FK monotherapy group, HSPs who received Basiliximab/FK+MMF+CS, were not at lower risk of death-censored allograft loss (p=0.55), rejection (p=0.11) or DSA (p=0.26).

Compared with the FK monotherapy group, HSPs who received Alemtuzumab/FK+MMF+CS, were not at lower risk of death-censored allograft loss (p=0.42), rejection (p=0.10) or DSA (p=0.11).

Compared with the FK monotherapy group, HSPs who received Alemtuzumab/FK+CS, were not at lower risk of death-censored allograft loss (p=0.47), rejection (p=0.16) or DSA (p=0.84).


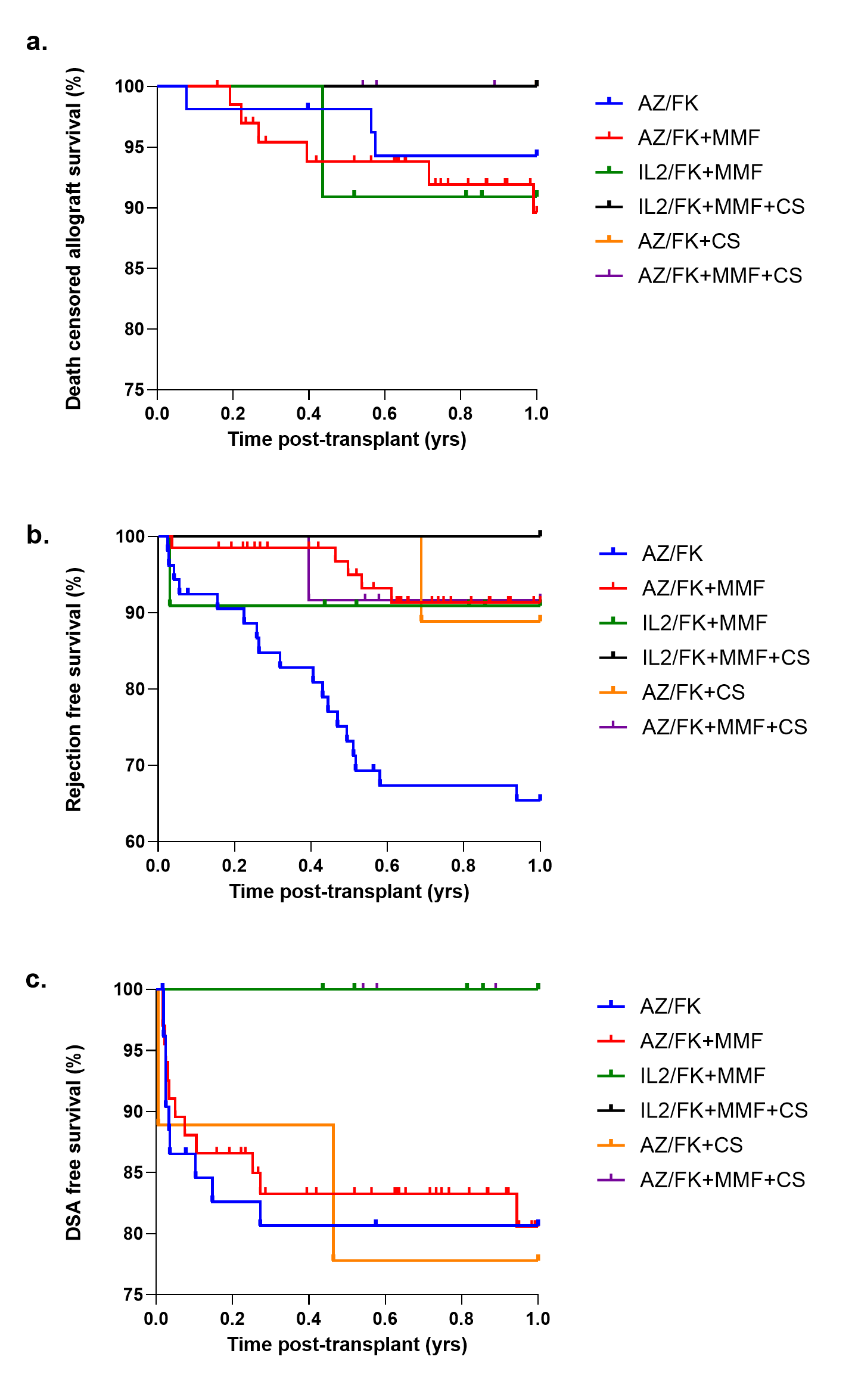

Supplement: Supplementary file 1 [file DataSheet1.docx]
